# CRISPR Interference–Potential Application in Retinal Disease

**DOI:** 10.3390/ijms21072329

**Published:** 2020-03-27

**Authors:** Caroline F. Peddle, Lewis E. Fry, Michelle E. McClements, Robert E. MacLaren

**Affiliations:** 1 Nuffield Laboratory of Ophthalmology, Nuffield Department of Clinical Neurosciences & NIHR Oxford Biomedical Research Centre, University of Oxford, Oxford OX3 9DU, UK; lewis.fry@ndcn.ox.ac.uk (L.E.F.); michelle.mcclements@eye.ox.ac.uk (M.E.M.); maclaren@eye.ox.ac.uk (R.E.M.); 2 Oxford Eye Hospital, Oxford University Hospitals NHS Foundation Trust, Oxford OX3 9DU, UK

**Keywords:** CRISPR interference, CRISPRi, CRISPR/Cas9, RNAi, knock-down, gene therapy, retinal disease, transcriptional repression, dCas9, KRAB

## Abstract

The treatment of dominantly inherited retinal diseases requires silencing of the pathogenic allele. RNA interference to suppress gene expression suffers from wide-spread off-target effects, while CRISPR-mediated gene disruption creates permanent changes in the genome. CRISPR interference uses a catalytically inactive ‘dead’ Cas9 directed by a guide RNA to block transcription of chosen genes without disrupting the DNA. It is highly specific and potentially reversible, increasing its safety profile as a therapy. Pre-clinical studies have demonstrated the versatility of CRISPR interference for gene silencing both in vivo and in ex vivo modification of iPSCs for transplantation. Applying CRISPR interference techniques for the treatment of autosomal dominant inherited retinal diseases is promising but there are few in vivo studies to date. This review details how CRISPR interference might be used to treat retinal diseases and addresses potential challenges for clinical translation.

## 1. Introduction

Inherited retinal diseases are an irreversible and devastating cause of blindness for an estimated 2 million individuals worldwide [1]. They are the result of genetic mutations that generally affect photoreceptors, the light-sensitive retinal cells at the back of the eye, typically causing progressive loss of sight over several years or decades. Over 250 individual genes have been implicated in inherited retinal disease, with autosomal recessive, autosomal dominant and X-linked inheritance patterns observed [2]. As most are monogenic disorders, gene therapy offers a potential treatment.

The eye has several advantages as a gene therapy site. It is easily accessible using ophthalmic surgical techniques allowing direct delivery to the target organ using local anaesthesia; it is immune privileged which reduces the likelihood of immune responses or rejection, and the presence of the blood retina barrier helps to prevent systemic spread of the introduced material [3,4,5]. These factors have driven dozens of gene therapy clinical trials, culminating in the first FDA approved ocular gene therapy treatment in 2017 [6,7].

**Figure 1:**
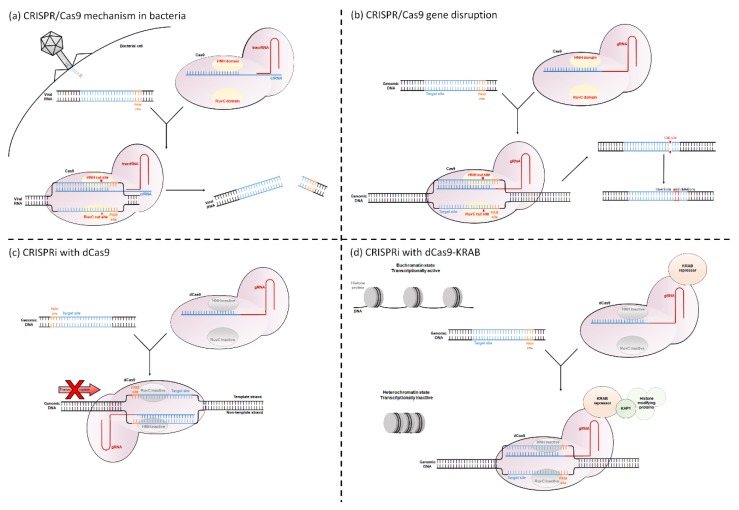
Mechanisms of CRISPR/Cas9 and CRISPRi. (**a**) CRISPR/Cas9 mechanism as a bacterial defence against invading viruses. (**b**) Using CRISPR/Cas9 to disrupt target genes. (**c**) Using CRISPRi with dCas9 to block transcription initiation or transcriptional elongation. (**d**) CRISPRi using a dCas9-KRAB fusion protein to alter the chromatin state of the gene.

Retinal gene therapy clinical trials have focused on treating recessive or X-linked loss-of-function mutations through gene augmentation, where a wild-type copy of a gene is introduced into affected cells. For successful treatment of dominant gain-of-function mutations, the pathogenic gene must be silenced [3,8]. Both the CRISPR/Cas9 (Clustered Regularly Interspaced Short Palindromic Repeats/CRISPR associated protein 9) system and CRISPR interference (CRISPRi) can be used to silence pathogenic genes. The CRISPR/Cas9 system is a naturally occurring bacterial defence mechanism against invading viruses. The bacteria contain two specialized RNA molecules called crRNA and tracrRNA, which form a complex with the endonuclease protein Cas9. This complex is guided to viral DNA that share sequence homology with crRNA. Cas9 requires a DNA element called a Protospacer Adjacent Motif (PAM) site immediately downstream of the crRNA binding sequence in order to bind to and cleave the DNA. The PAM site is a short nucleotide sequence (usually 3 to 8 bp long), the exact sequence requirements of which vary with bacterial species (for example *Streptococcus pyogenes* Cas9 has the PAM site 5′-NGG-3′). Following binding, Cas9 will cleave both strands, preventing viral colonization [9,10,11,12] (Figure 1a).

This system is used within biological research to disrupt genes of interest. The target gene is screened for PAM sites, and then a single guide RNA (gRNA, which is an artificial amalgamation that links both the crRNA and tracrRNA) is designed to match the region immediately upstream of this. By providing the cell with both the gRNA and Cas9, Cas9 will induce a double stranded break in the target gene. The cell then attempts to repair this break, but in the absence of a DNA template, this process is extremely error prone. This causes small insertions and deletions to be incorporated at the DNA break site, resulting in frame shifts or premature stop codons within the DNA [10,13] (Figure 1b).

CRISPRi uses a modified version of the Cas9 protein. The native Cas9 protein contains two DNA cleavage domains called HNH and RuvC, which cleave the gRNA complementary and gRNA non-complementary DNA strands, respectively [14]. By changing a single amino acid at each domain (for the *Streptococcus pyogenes* Cas9, the amino acid changes are D10A and H840A [15]), the Cas9 becomes catalytically inactive. This “dead” dCas9 can still bind to DNA but can no longer cleave it. The dCas9 causes transcriptional repression of the target gene. This is either through blocking the passage of RNA polymerase or through a dCas9-fusion protein altering the histone state of the gene (See Opportunities for Gene Regulation in Eukaryotes and Mechanisms of CRISPRi) (Figure 1c–d).

This review will outline the technique of CRISPRi, how it might be applied to treat dominantly inherited retinal dystrophies, and discuss important considerations for its clinical use.

## 2. Opportunities for Gene Regulation in Eukaryotes

The regulation of gene expression within eukaryotes is complex and occurs at multiple levels within the cell. Each of these levels offers an opportunity for expression to be manipulated with molecular techniques (see Table 1). The following is a brief introduction to the main regulatory systems in eukaryotic cells that can be targeted with interference methods.

Regulation begins at a DNA level, where cells can alter the rate of transcription from the gene’s promoter. Transcription initiation varies between genes, but generally, transcription factors form a complex with RNA polymerase II, which allows it to bind to the transcriptional start site (TSS) [16]. Following binding, the interaction with distal regulatory elements dictates whether transcription will begin [17,18].

During early development of multi-cellular organisms, promoters can be permanently inactivated via an epigenetic mechanism called DNA methylation. Regions of DNA called “CpG islands” are common in eukaryotic promoters and contain repetitive sequences of 5′-CG-3′. DNA methyltransferase adds a methyl group to cytosines, which results in permanent silencing of that gene, which is inherited by daughter cells [19].

Following successful transcription of the DNA and subsequent processing, mRNA is translated into an amino acid chain. The lifespan of the mRNA molecule determines how much protein is produced (a longer half-life produces more protein), and cells employ a range of mechanisms to trigger or prevent degradation of the mRNA molecule. RNA interference (RNAi) acts to either block translation or trigger early degradation of the mRNA [20]. In RNAi, short double stranded micro-RNAs are produced that are complementary to the target mRNA. Micro-RNAs associate with the RNA-induced silencing complex and bind to the target mRNA, which is then either cleaved and rapidly degraded, or has its translation inhibited. This results in gene suppression.

The final level of gene regulation is at a histone level. Within the nucleus of a cell, the DNA is wound around histone proteins. In an open confirmation (euchromatin), the genes are active. In a tightly wound state (heterochromatin), transcription factors and RNA polymerase II cannot access the genes, making them inactive. The histone proteins have tails that can be chemically “tagged”, changing their confirmation and therefore altering DNA accessibility. Transcription factors recruit histone modifying proteins, such as histone acetyltransferase or histone deacetylase, altering the transcription rate of the gene [21].

## 3. Mechanisms of CRISPRi

Targeting a genetic locus with CRISPRi requires only three main components. The first component is a PAM site in the DNA target region. The second component is a gRNA, which is designed with a region that is homologous to the area immediately 5′ adjacent to the chosen PAM site. The third component is the dCas9 protein, which forms a complex with the gRNA. The gRNA portion of the dCas9:gRNA complex will bind to the target DNA via sequence homology while dCas9 binds to the adjacent PAM site [10].

The basic CRISPRi mechanism uses the dCas9 protein to sterically block key regions of the target gene, preventing access of crucial transcriptional proteins. This reduces transcription and therefore downregulates expression. CRISPRi is either targeted to the TSS to prevent initiation of transcription or to downstream transcribed regions to block transcriptional elongation [15,22] (Figure 1c).

When blocking transcription initiation, either strand of DNA can be targeted. Large-scale analysis studies have identified the optimal binding site for blocking transcription initiation using CRISPRi as −50 to +250 base pairs relative to the TSS [23,24].

During transcriptional elongation, RNA polymerase II unwinds double stranded DNA and uses the template strand (also called the non-coding strand, reverse strand, or anti-sense strand) to create mRNA. gRNAs targeted to the template strand have reduced knock-down [15,22,25], although knock-down targeting this strand has been observed [23,26]. This could be due to RNA polymerase II unwinding the gRNA:DNA complex as they are “facing” each other. When the gRNA is targeted to the non-template strand of DNA the dCas9:gRNA complex acts as a physical barrier to the passage of RNA polymerase II, preventing further transcription [15,22]. Unlike targeting the TSS, there is currently no consensus on which regions of DNA are optimal and there is high variability between genes [24,27].

To increase the impact of CRISPRi, a 2013 study fused dCas9 with a range of transcriptional repressor domains [25]. These domains are typically eukaryotic protein domains which regulate transcription. The Krüppel Associated Box (KRAB) repressor is most commonly used. It interacts with the scaffold protein KAP1, which recruits histone modifying proteins, ultimately creating a tightly-wound, inactive heterochromatin histone state [28] (Figure 1d). Gilbert et al. found that addition of KRAB significantly increased the repression of genes at most tested loci, with one site reducing the ubiquitous EGFP expression by 15-fold in a GFP+HEK293 cell line [25]. Unlike when using dCas9 alone to block transcriptional elongation, dCas9-KRAB can be targeted to either strand of the DNA, dramatically increasing the potential target sites [27].

Although not typically defined as CRISPRi, it is worth noting that dCas9 can be fused to various proteins, which alter the epigenome of the cell and can result in down- or up- regulation of target genes. dCas9 has been successfully fused to proteins which alter the chemical tags on histone molecules: fusion to methyltransferase domains inactivates genes, and fusion to proteins, which remove methyl groups, activates repressed genes [29,30]. For a recent review on this, see [29].

## 4. Alternatives to CRISPRi

### 4.1. CRISPRi vs. CRISPR/Cas9

CRISPR/Cas9 causes permanent changes to the genome, whereas CRISPRi does not. After binding to DNA, Cas9 cleaves it, introducing small insertions and deletions into the target site, which alter the amino acid sequence and cause premature stop codons [10]. CRISPRi, on the other hand, does not alter the DNA sequence and will only affect transcription while dCas9 and gRNA are present. Experiments using inducible promoters have demonstrated that the effects of CRISPRi can be reversed following termination of dCas9 expression. In mRFP-expressing *E. coli* cells transduced with a plasmid carrying dCas9, suppressed mRFP fluorescence levels returned to baseline 300 min after switching off dCas9 expression [15]. In eukaryotic K562 cell lines stably transduced with dCas9-KRAB, target mRNA and protein expression returned to baseline 6 days after switching off dCas9-KRAB expression [23]. This provides safety benefits for gene therapies as CRISPRi treatment could be reversed if driven with an inducible promoter.

CRISPR/Cas9 and CRISPRi have different optimal target requirements. CRISPR/Cas9 is most successful when targeted to the exon of a gene, as insertions and deletions in introns can be removed through splicing leaving an unaltered coding sequence [31]. CRISPRi, on the other hand, can in theory be targeted anywhere in the transcribed region. In practice, however, the optimal target site for CRISPRi is quite narrow, at −50 to +250 relative to the TSS [23,24]. If there is a particular locus of the gene that is being targeted, it may only be possible using either CRISPR/Cas9 or CRISPRi.

While CRISPRi can be highly efficient for some targets, with one paper reporting a 1000-fold repression of the mRNA target, it is generally accepted that the active cutting form of CRISPR/Cas9 is more efficient at knocking down target gene expression than CRISPRi [15,27,32]. This is likely because the efficiency of CRISPRi gene repression is not only dictated by the gRNA:DNA binding, as with active Cas9, but by the ability of that locus to successfully block transcription [23]. This makes the knock-down rates seen with CRISPRi highly variable across the sequence of a gene [15,23,24].

CRISPRi may have greater specificity than CRISPR/Cas9. Both systems rely on sequence homology between their gRNA and DNA, and the presence of an immediately adjacent PAM site to bind. Off-target binding occurs when DNA with partial homology to the gRNA sequence is adjacent to a PAM site. CRISPR/Cas9 has variable tolerance for mismatches between its gRNA and target DNA sequence and off-target effects are common; some have been reported at sites with a 5 bp gRNA:DNA mismatch [10,33,34,35,36]. This has led to the development of “high-fidelity” Cas9 species, which are engineered to have reduced off-target activity; in some cases, they have no detectable off-target effects [37,38,39,40]. CRISPRi, on the other hand, has very low tolerance for gRNA:DNA mismatches [23]. One paper reported that a 1 bp mismatch at every gRNA position prevented CRISPRi activity with their tested gRNA (although they noted a trend of gRNA:DNA mismatches at sites distal to the PAM being better tolerated, as observed for active Cas9) [9,10,34,35,41]. Another paper reported that a single gRNA:DNA mismatch significantly reduced the knock-down rate at every gRNA position, while a combination of two or more mismatches was enough to abolish knock-down entirely [23]. This might be due to the different mechanisms between CRISPR/Cas9 and CRISPRi: CRISPR/Cas9 may be able to cleave DNA whilst in contact with it only briefly, which might be the case with mismatched nucleotides, whereas for CRISPRi to be effective, it is likely to need to be in contact with the DNA target for considerably longer. In support of this notion, genome-wide analyses of the effects of CRISPRi commonly report no detected off-target effects on the cell’s transcriptome [15,25,42] (see Table 2).

### 4.2. CRISPRi vs. RNAi

Until the creation of inactive dCas9, RNAi was the leading technique for suppressing gene expression in eukaryotic cells. It utilizes the cells natural RNAi pathway (see Opportunities for Gene Regulation in Eukaryotes). In RNAi, a short RNA sequence complementary to the target is introduced to the cell. It associates with the RNA-induced silencing complex and binds to the target mRNA via sequence homology. This triggers a pathway that will cause either mRNA degradation or a reduction in translation [20].

RNAi is arguably a simpler technique than CRISPRi, requiring only successful cellular delivery of a short double stranded RNA molecule. In ocular diseases, chemically modified RNA molecules are delivered directly to the eye without a delivery vehicle [43]. It has been used for gene knock-down since 2001 and is well characterized, with numerous therapies in clinical trials (including treatments for ocular diseases such as age-related macular degeneration, non-arteritic anterior ischaemic optic neuropathy, and dry eye disease) and two FDA-approved treatments for hereditary transthyretin-mediated amyloidosis and acute hepatic porphyria [43,44].

While RNAi reduces translation, CRISPRi reduces transcription. As it binds to DNA, rather than mRNA, the CRISPRi system has access to more potential binding sites in the untranscribed regions of DNA, such as the promoter and distal regulatory elements. It can also be targeted to introns, which are not accessible to RNAi as it generally targets mRNA [42].

Efficiency and specificity of the treatment strategies have been compared. Off-target effects of CRISPRi are rare, with multiple studies reporting no significant off-target effects detectable with RNA-Seq [23,25,27,41]. With RNAi on the other hand, off-target effects are commonly reported [15,45,46,47]. Wide-scale studies have identified that CRISPRi and RNAi have comparable efficiencies, but these are highly variable and target-specific [46]. At an individual gene level, CRISPRi is often able to outperform RNAi, with one study achieving CRISPRi-mediated protein knock-down of almost 90% for the 4 tested genes, compared to more variable knock-down with RNAi [41] (see Table 2).

## 5. Clinical Treatment Strategies

CRISPRi allows highly specific and efficient gene repression and is therefore a promising clinical treatment if targeted to genes involved in disease pathology. This has high applicability in retinal diseases but there is currently only one published in vivo study targeting the retina [48]. The two main therapeutic strategies being explored across diseases are in vivo knock-down of a target gene, in which the CRISPRi construct is applied directly to the target organ, and ex vivo knock-down, where CRISPRi is applied to cells, which are then transplanted into a patient. Pre-clinical studies in animal models using CRISPRi to target human disease are outlined in Table 3. CRISPRi has also proven a useful tool for biological research in vivo for creating knock-down mouse models [49] and identifying gene function [41,50], but these are not discussed here.

### 5.1. In Vivo Knock-Down

CRISPRi can be used to knock-down expression of genes involved in retinal disease by delivering the construct with gene therapy via a subretinal injection.

#### 5.1.1. Targeting the Pathogenic Mutation

For diseases caused by dominant gain-of-function mutations, such as retinitis pigmentosa caused by rhodopsin mutations, CRISPRi must be targeted exclusively to the pathogenic allele. This can be done in two ways: targeting the pathogenic mutation directly or targeting a single nucleotide polymorphism (SNP) on the same allele as the pathogenic mutation. The mutation or SNP must either generate a novel PAM site or be in the gRNA binding region. Generating a PAM site not present on the non-targeted allele allows the gRNA:dCas9 complex to bind specifically to the target allele. A mutation or SNP in the binding region of the gRNA creates a mismatch of at least 1 bp with the wild type strand. As discussed in “CRISPRi vs. CRISPR/Cas9”, CRISPRi is extremely sensitive to mismatches between its gRNA and the target DNA, with 1 bp mismatches often sufficient to prevent dCas9 binding and therefore gene repression [23,41].

Allele-specific targeting of the pathogenic mutation has been used to treat in vivo models of autosomal dominant retinitis pigmentosa with active CRISPR/Cas9. Bakondi et al. and Li et al. targeted pathogenic mutations in *Rho* occurring in the PAM site and gRNA binding region, respectively. In both cases, only the pathogenic allele was disrupted, resulting in significantly improved visual outcomes [51,52]. Bakondi et al. measured increased retinal preservation and a 35% improvement in optokinetic response, a surrogate measurement for visual function, in the treated eye of S334ter-3 rats compared to the untreated eye. Li et al. found significantly more photoreceptor cells in the CRISPR-treated retinal areas of P23H mice, indicating the retinal degeneration was slowed.

#### 5.1.2. Cellular Reprogramming

Cellular reprogramming aims to convert a diseased cell type into a cell type that is unaffected by the pathogenic mutation [53]. This was potentially demonstrated with CRISPRi to treat autosomal recessive retinitis pigmentosa in the Rd10 mouse model. Rd10 mice contain a mutation in the *Pde6β* gene, which causes complete rod cell death by P60 but allows relative preservation of cone cells. Moreno et al. targeted the rod cell transcription factor *Nrl,* which is a regulator determining rod cell fate over cone cell fate in photoreceptors. The treated mice developed a more cone-like phenotype, which was associated with increased photoreceptor cell layers and visual acuity [48]. While patients treated in this way would lose vision characteristics driven by rod cells, such as night vision, relative preservation of cone cells may allow patients to maintain visual acuity and central vision.

#### 5.1.3. Treating Disease Pathways

A final strategy for in vivo knock-down is targeting genes that drive damaging disease pathways, therefore slowing the disease progression. This technique is useful for polygenic conditions where targeting a single pathogenic gene is not possible.

CRISPRi has been used in this way to treat familial hypercholesterolemia and obesity in mouse models in vivo by knocking down genes that are involved in the disease pathology. In treating familial hypercholesterolemia, the gene *Pcsk9,* which functions in the regulation of low-density lipoprotein receptor degradation, was targeted. Over 80% reduction in PCSK9 protein was achieved in the liver, and a significant reduction in serum low-density lipoproteins was found in the treated mice [54]. For the treatment of obesity, the expression of lipid chaperone protein *Fabp4* was significantly reduced in the high-fat diet-induced mice, which lead to a reduction in body weight, fat mass, and blood glucose levels in the CRISPRi-treated mice [26].

Wet age-related macular degeneration can lead to the loss of the central visual field due to the development of choroidal neovascularization. Current treatment of wet age-related macular degeneration uses drugs to block growth factors that contribute to this disease pathway, such as vascular endothelial growth factor. Disruption of this pathway using RNAi and CRISPR/Cas9 has been demonstrated in principle, achieving potentially significant therapeutic effects, and similarly, CRISPRi could be used to knock-down key genes [55,56].

### 5.2. Ex Vivo Knock-Down

As well as downregulating a target gene in vivo, CRISPRi has demonstrated effectiveness at suppressing gene function in stem cell-derived cells for implantation into patients [57]. In a paper aiming to improve the low rates of calvarial bone healing, CRISPRi was used alongside CRISPR activation to repress and activate genes involved in the inhibition and promotion of cartilage formation, respectively. dCas9-KRAB reduced its target *PPAR-γ* expression by 30.3% in bone marrow-derived mesenchymal stem cells. The cells were then cultured into engineered cartilage and implanted into calvarial bone defects in rats. The CRISPR-untreated cartilage had negligible bone growth, while the CRISPR-treated cartilage saw significant bone repair, filling 28.6% and 23.3% of the original defect area [58].

This method has application in retinal diseases, where late stage patients have wide-spread loss of retinal cells causing significant gaps in their field of vision. In cases of autologous transplantation, CRISPRi could be used to suppress the pathogenic gene in induced pluripotent stem cells (iPSCs) pre-implantation before reprogramming into the desired retinal cell type, and autologous transplantation. The potential of this treatment has been demonstrated with successful CRISPR/Cas9 gene correction of pathogenic retinal mutations in iPSCs for retinitis pigmentosa and Leber congenital amaurosis [59,60,61]. Following correction of three different *RPGR* frame shift mutations in patient iPSCs, Deng et al. cultured the iPSCs into retinal organoids and found that the X-linked retinitis pigmentosa phenotype was completely alleviated [60].

Clinical studies using stem cell-derived cells for the treatment of inherited retinal disease are currently underway. Among them, Stargardt disease and retinitis pigmentosa are being investigated, with stem-cell-derived retinal pigment epithelium cells, and retinal progenitor cells, respectively, injected into patient’s eyes. Initial results have documented good tolerance of the introduced cells, with some indications of symptom improvement, although full results are not yet published [62].

## 6. Challenges to Clinical Application

### 6.1. Target Selection and Efficacy

As covered in “CRISPRi vs. RNAi”, optimally designed CRISPRi has comparable efficiency to RNAi, the technique previously favored to knock-down clinically relevant genes [46]. Unfortunately, CRISPRi knock-down is highly variable throughout the gene as not all regions will block transcription effectively [15]. Outside the optimal −50 to +250 (relative to TSS) target region, one paper found only 56% of gRNAs achieved knock-down of over 20% [24]. While the −50 to +250 region gives plenty of potential target sites for a knock-down and replace strategy, it is relatively limited if the strategy involves targeting a specific region of the gene, for example targeting the pathogenic mutation for allele-specific knock-down.

Improvements have been made to methods for predicting successful target sites. The FANTOM5/CAGE atlas is a database annotating regulatory elements of mammalian genomes. By identifying the transcriptional start site using this software and then using traditional CRISPR software to analyse the gRNA:DNA binding success, gRNA efficiency predictions were significantly improved [24].

Gene repression rates can often be increased with the addition of the KRAB repressor [25,27]. A triple fusion protein of dCas9, KRAB repressor, and MeCP2 repressor domain (which initiates chromatin remodelling via a different pathway from the KRAB repressor) was found to further increase gene repression [27].

When targeting pathogenic gain-of-function mutations, it is useful to note that wild type phenotypes can often be restored with less than 100% knock-down of the mutant gene. In Bakondi et al. 2016, for example, retinal morphology was significantly improved in a rat model of autosomal dominant retinitis pigmentosa with CRISPR/Cas9 editing rates of 33% and 36%. Photoreceptor layers were increased from 1 layer in the control group to 8 layers in the CRISPR-treated group [51]. Even partial reduction in the expression of toxic genes may slow the rate of degeneration and result in preservation of visual function and delay of vision loss [53,55,63,64].

### 6.2. Delivery to the Retina

Once a CRISPRi construct is created, the next challenge is successfully delivering it to the target cell. Clinical retinal gene therapy trials to date have used Adeno-Associated Virus (AAV). AAV vectors are non-integrating and offer strong expression, an excellent safety profile, and the ability to effectively transduce photoreceptors and RPE when subretinally injected [65]. The main limitation of AAV is its roughly 4.7 kb DNA packaging capacity. Cas9s have a relatively large coding sequence and in vivo experiments using the traditionally favoured 4104 bp SpCas9 have adopted a dual vector approach to overcome this [66]. Multiple shorter variants of Cas9 have been identified which can be packaged into a single AAV along with their regulatory elements, and a gRNA [55,67,68,69]. In particular, Cas9 species from *Staphylococcus aureus*, *Streptococcus thermophiles*, *Neisseria meningitides*, and *Campylobacter jejuni* are all under 3.5 kb, and their inactive dCas9 form has been used in vitro experiments [70]. The 3159 bp *Staphylococcus aureus* Cas9 has also been used in in vivo CRISPRi studies and successfully reduced target protein expression by 80% [48].

Lentiviruses are integrating vectors with high efficiency of transgene expression. Unfortunately, efficient transduction of photoreceptors is currently limited to newborn animals [7,71,72]. The integration site is also difficult to predict, which raises concerns of off-target effects and potential oncogenesis. For these reasons, lentivirus is usually unsuitable for in vivo knock-down but is frequently used for ex vivo knock-down, where all potential off-target effects can be screened before the cells are transplanted into the patient.

Non-viral delivery methods include ribonucleoproteins (RNPs) or plasmid DNA. RNPs consist of a Cas9 protein bound to a gRNA, which is delivered to cells through lipid-based transfection or electroporation. While RNPs have successfully induced CRISPR-mediated gene disruption in mouse RPE cells, none of the neural retina took up the RNP successfully [73]. The turnover rate of RNP in the eye was very high, with all Cas9 protein degraded 3 days after injection, making it unsuitable for CRISPRi delivery. Multiple studies have used plasmid electroporation to deliver CRISPR/Cas9 into newborn rat retinas with successful transduction in multiple retinal layers [51,74,75]. Despite this, therapeutic application is limited as delivery to post-mitotic cells (as in adult retinas) appears to be less efficient [72].

### 6.3. Immune Response

Immune responses to gene therapy are a concern in the field. Reactions to the transgene, transgene product or the packaging method can cause inflammation, reduced efficacy, and pose health risks to patients. Advantageously, the eye benefits from immune privilege. The blood-ocular barrier allows the eye to function as a separate microenvironment, altering immune cell activity, suppressing inflammation, and improving tolerance of introduced foreign elements [76]. Unfortunately, this system is not impenetrable, and breakdown of the blood-ocular barrier can introduce adaptive immune cells to the eye [77]. Intraocular inflammation has been reported in multiple AAV gene therapy trials, and therefore, the potential immune response to introduced CRISPRi elements must be considered [78,79,80].

The two most popular Cas9 species are from pathogenic bacteria: SaCas9 from *Staphylococcus aureus* and SpCas9 from *Streptococcus pyogenes*. It is therefore unsurprising that antibodies and cytotoxic T cells against SaCas9 and SpCas9 have been documented in healthy humans [81,82,83]. If activated, these could destroy Cas9 proteins found extracellularly or kill cells expressing Cas9 internally.

Immune responses also occur against gRNAs that are transcribed in vitro and used in RNPs. These gRNAs contain a 5′-triphosphate group which is associated with viruses and triggers the type-I interferon-mediated innate immune response within the cell. In one study, this caused cytotoxicity of both the treated and surrounding untreated cells in vitro but was completed alleviated by chemically converting the viral 5′-triphosphate group to a 5′-hydroxyl group [84].

No immune response experiments have been conducted with dCas9, although the response is assumed to be similar to that from Cas9. Successful CRISPRi requires long term stable expression of dCas9, which gives a wide window for potentially dangerous immune responses to occur, so this area must be researched thoroughly.

## 7. Conclusions

CRISPRi is a promising tool for the treatment of inherited retinal diseases. CRISPRi has a strong safety profile, as off-target repression is extremely rare, and the effects are completely reversed upon degradation of dCas9 or gRNA. Its low tolerance of gRNA:DNA mismatches makes it especially useful for allele-specific knock-down of dominant toxic gain-of-function mutations. While its efficiency can be highly variable, strong gene repression is achievable, and advances in fusion protein modifications and target site predictions are improving experimental outcomes.

## Figures and Tables

**Table 1:** Molecular methods used to target different gene regulation systems in eukaryotes.

Gene Regulation	Method
Gene transcription	CRISPRi
DNA methylation	dCas9-methyltransferase
mRNA lifespan/gene translation	RNAi
Histone state	CRISPRi

**Table 2:** Comparison of CRISPRi, CRISPR/Cas9 and RNAi methods for knock-down of eukaryotic genes. + indicates lower levels, ++ indicates higher levels.

Feature	CRISPRi	CRISPR/Cas9	RNAi
Target	DNA	DNA	mRNA
Requirements	gRNA complementary to target. dCas9 protein	gRNA complementary to target. Cas9 protein	Short interfering RNA complementary to target
Efficiency	+	++	+
Specificity	++	+	+

**Table 3:** In vivo gene therapy CRISPRi studies.

Reference	Treatment Method	Condition	gRNA Target	Experimental Methodology	Results
Moreno et al. 2018 [48]	In vivo knock-down Cellular reprogramming	Autosomal recessive retinitis pigmentosa	*Nrl*	Dual subretinal injection of AAV.gRNA.dSpCas9-KRAB.N-terminus and AAV.dSpCas9-KRAB C-terminus into Rd10 mouse.	Rods developed a more “cone-like” phenotype. Increased photoreceptor layer thickness. Significantly improved visual function.
Thakore et al. 2018 [54]	In vivo knock-down Treating disease pathways	High LDL cholesterol (familial hypercholesterolemia)	*Pcsk9*	Dual injection of AAV.dSaCas9-KRAB and AAV.gRNA into mouse tail vein.	80% reduction in target protein. Significant reduction in serum LDL cholesterol.
Chung et al. 2019 [26]	In vivo knock-down Treating disease pathways	Obesity	*Fabp4*	Intraperitoneal injection of ATS-9R peptide and dSpCas9.gRNA plasmid oligoplex into HFD-induced obesity and diabetes model mice.	Significant reduction in target mRNA. Improved disease symptoms including decrease in body weight, fat mass, and blood glucose.
Yoshida et al. 2018 [57]	In vivo knock-down Treating disease pathways (Experimental methodology required ex vivo knock-down)	Lung squamous cell carcinoma	∆Np63	Lentiviral delivery of dSpCas9-KRAB.gRNA to EBC2 lung SCC cells. Xenograft then injected into adult mice.	Tumour growth significantly repressed
Truong et al. 2019 [58]	Ex vivo knock-down	Calvarial bone healing	*PPAR-γ* (repression) and *Sox9* (activation)	Bacilloviral delivery of all-in-one CRISPRai construct into rat bone marrow-derived mesenchymal stem cells. These were implanted into rat calvarial bone defects.	Significant increase in calvarial bone healing.

## Abbreviations

AAV	Adeno-associated virus
CRISPRi	CRISPR interference
gRNA	Guide RNA
iPSCs	Induced pluripotent stem cells
KRAB	Krüppel associated box
RNAi	RNA interference
RNP	Ribonucleoprotein
SNP	Single nucleotide polymorphism
TSS	Transcriptional start site
